# Alternative processing of its precursor is related to miR319 decreasing in melon plants exposed to cold

**DOI:** 10.1038/s41598-018-34012-7

**Published:** 2018-10-19

**Authors:** Antonio Bustamante, Maria Carmen Marques, Alejandro Sanz-Carbonell, Jose Miguel Mulet, Gustavo Gomez

**Affiliations:** 1 Institute for Integrative Systems Biology (I2SysBio), Consejo Superior de Investigaciones Científicas-Universitat de Valencia (CSIC-UV), Parc Cientific UV, Catedratico Agustin Escardino 9, 46980 Paterna, Spain; 2 Instituto de Biología Molecular y Celular de Plantas (IBMCP), Consejo Superior de Investigaciones Científicas (CSIC)-Universitat Politecnica de Valencia (UPV), CPI, Edificio 8 E, Av. de los Naranjos s/n, 46022 Valencia, Spain; 3 Instituto Nacional de Investigaciones Agropecuarias (INIAP), Estación Experimental Pichilingue, Km5 vía Quevedo El Empalme, Mocache, Ecuador

**Keywords:** Melon Plants, Cold-induced Stress, Rapid Amplification Of cDNA Ends (RACE), Partial Cleavage, miRNAs, Plant stress responses, Abiotic

## Abstract

miRNAs are fundamental endogenous regulators of gene expression in higher organisms. miRNAs modulate multiple biological processes in plants. Consequently, miRNA accumulation is strictly controlled through miRNA precursor accumulation and processing. Members of the miRNA319 family are ancient ribo-regulators that are essential for plant development and stress responses and exhibit an unusual biogenesis that is characterized by multiple processing of their precursors. The significance of the high conservation of these non-canonical biogenesis pathways remains unknown. Here, we analyze data obtained by massive sRNA sequencing and 5′ - RACE to explore the accumulation and infer the processing of members of the miR319 family in melon plants exposed to adverse environmental conditions. Sequence data showed that miR319c was down regulated in response to low temperature. However, the level of its precursor was increased by cold, indicating that miR319c accumulation is not related to the stem loop levels. Furthermore, we found that a decrease in miR319c was inversely correlated with the stable accumulation of an alternative miRNA (#miR319c) derived from multiple processing of the miR319c precursor. Interestingly, the alternative accumulation of miR319c and #miR319c was associated with an additional and non-canonical partial cleavage of the miR319c precursor during its loop-to-base-processing. Analysis of the transcriptional activity showed that miR319c negatively regulated the accumulation of HY5 via TCP2 in melon plants exposed to cold, supporting its involvement in the low temperature signaling pathway associated with anthocyanin biosynthesis. Our results provide new insights regarding the versatility of plant miRNA processing and the mechanisms regulating them as well as the hypothetical mechanism for the response to cold-induced stress in melon, which is based on the alternative regulation of miRNA biogenesis.

## Introduction

MicroRNAs (miRNAs) are a class of endogenous small RNAs (usually 20- to 22-nt long) that act as components of the RNA silencing machinery and play a fundamental role in controlling gene expression in most eukaryotic organisms. In plants, miRNAs regulate diverse biological processes, such as development, metabolism, senescence and the stress response^1–3^. miRNAs are generated from an RNA polymerase II-derived primary transcript (pri-miRNA) that includes a foldback structure with mature miRNAs located in one of the arms^4^. miRNA processing is achieved by the nuclear RNAse DICER-LIKE 1 (DCL1) and its accessory proteins SERRATE (SE) and HYPONASTIC LEAVES 1 (HYL1), which increase cleavage efficiency and accuracy^5,6^. The precision of the position of the cuts along the precursor is important, as they determine the definitive miRNA/miRNA* sequence and, consequently, its target specificity^7^. The resulting miRNA/miRNA* duplex is then 2′*O*-methylated at both 3′ ends by HUA1 ENHANCER (HEN1), a small RNA methyltransferase that interacts with the DCL1 complex^8^ and is exported into the cytoplasm. Once loaded into the RNA Induced Silencing Complex (RISC), which contains a member of the ARGONAUTE family (usually AGO1) as a main component, miRNAs anneal to complementary mRNAs to modulate their target expression by cleavage and/or translational inhibition^5,9^. Taking into account that miRNAs are key components of the gene expression regulatory network, their accumulation in plants is strictly and predominantly controlled through pri-miRNA accumulation and processing^9^. Whereas the pri-miRNA levels depend on their stability (interplay between transcription and degradation), processing may be affected by the stem-loop structure and length^7^ and/or the action of accessory proteins^10^. This fine modulation of miRNA biogenesis is crucial for plant development and adaptation to environmental changes^9^.

The hairpins of plant miRNA precursors have diverse structures and lengths that range from 50 to >500 nt^11^; this heterogeneity may also reflect significant differences in their processing^7^. A single stem loop usually harbors a unique miRNA/miRNA* duplex; however, some particularly long stem loops can give rise to alternative sRNAs besides the expected miRNAs^12^. The precursors of miR159 and miR319 have non-canonical hairpin structures and processing. Diverse studies have shown that during the processing of members of the miR159 and miR319 families, DCL1 cuts their precursors at four different points to release additional miRNA/miRNA* duplexes that can be occasionally detected *in vivo* ^13–17^. A detailed mutagenesis analysis revealed that miR319 could not be efficiently excised from its stem loop if this multiple processing was impaired^15^. The evolutionary conservation of this biogenesis pattern is inexplicable according to the known function of miR159/319; consequently, the biological significance of this intriguing processing remains unknown^16^.

MiR159 and miR319 are highly conserved ribo-regulators that play a fundamental role in plant development^16^. Although both exhibit high sequence similarity, miR159 and miR319 have differential expression patterns and regulate distinct targets. The miR159 family (highly and ubiquitously accumulated in plants) regulates the expression of MYB transcription factors^18,19^, while miR319 members (with tissue-specificity and lower expression) target a group of TCP transcription factors that regulate plant cell proliferation^20,21^. Interestingly, a close interaction between miR319 accumulation and the response to cold-induced stress has been extensively reported in diverse plant species, such as rice^22^, sugarcane^23^ arabidopsis^24^, and tomato^25^.

Melon (*Cucumis melo* L.) is a eudicot diploid plant species (2n = 2x = 24) that is of interest because of its specific biological properties and economic importance^26^. Although melon is one of the more extensively cultivated members of the family *Cucurbitaceae*, several environmental factors seriously affect melon crop production^27^. Different studies have contributed to identifying the diverse miRNAs that are involved in the stress-response in melon plants^28–30^. However, little is known about how miRNA biogenesis is affected by adverse environmental conditions in this economically important crop.

Here, we show that selective alternative processing of the miR319 family occurs in melon plants exposed to low temperature conditions. The results obtained from sRNA sequencing showed that although all miR319 members were significantly downregulated in response to cold, one of them, miR319c, demonstrated lower accumulation under this stress condition. By contrast, its precursor level was drastically increased by cold, indicating that miR319c accumulation is not related to stem loop transcription and/or stability. The levels of the miR319c population were inversely correlated with the accumulation of an alternative miRNA (#miR319c) derived from non-canonical processing of the miR319c precursor. Interestingly, this alternative accumulation of miR319c and #miR319c was associated with an unexpected partial cleavage of the miR319c precursor during its loop-to-base-processing, indicating that this dual pri-miRNA processing represents a phenomenon associated with the regulation of miRNA319c biogenesis under low temperature. These results provide new insights into the versatility of plant miRNA processing and the mechanisms regulating them as well as a new pathway for responding to cold stress in melon based on an alternative regulation of miRNA biogenesis.

## Results

### Cold treatment downregulates the cme-miR319 family

In this study, we analyzed data obtained from differential expression analysis of high-throughput sequencing of small RNA libraries constructed from melon plants exposed to cold for 11 days and non-treated plants used as controls. Correlations between sRNA expression profiles were estimated by principal component analysis (PCA) and *Pearson* correlation. The obtained results showed that the biological replicates with the same condition were clustered together and clearly distinguishable from the control, attesting to the reproducibility of our assay (Figure S1). Reads fully homologous to rRNA, tRNA, snoRNA and snRNA sequences deposited in the Rfam data base (http://rfam.xfam.org), representing the 2.17% and 3.4% of the sequenced sRNAs in control and cold treated samples, respectively, were filtered out. The sRNA abundance was statistically analyzed with the edgeR-Bioconductor package. Reads with significant differential expression were identified based on two criteria: a log2 Fold Change (log2FC) >2.0 or <−2.0 and a False Discovery Rate (FDR) value ≤ 0.05. Only reads that were fully homologous to the previously described members of the cme-miR319 family deposited in miRBase were included in further analyses.

**Figure 1:**
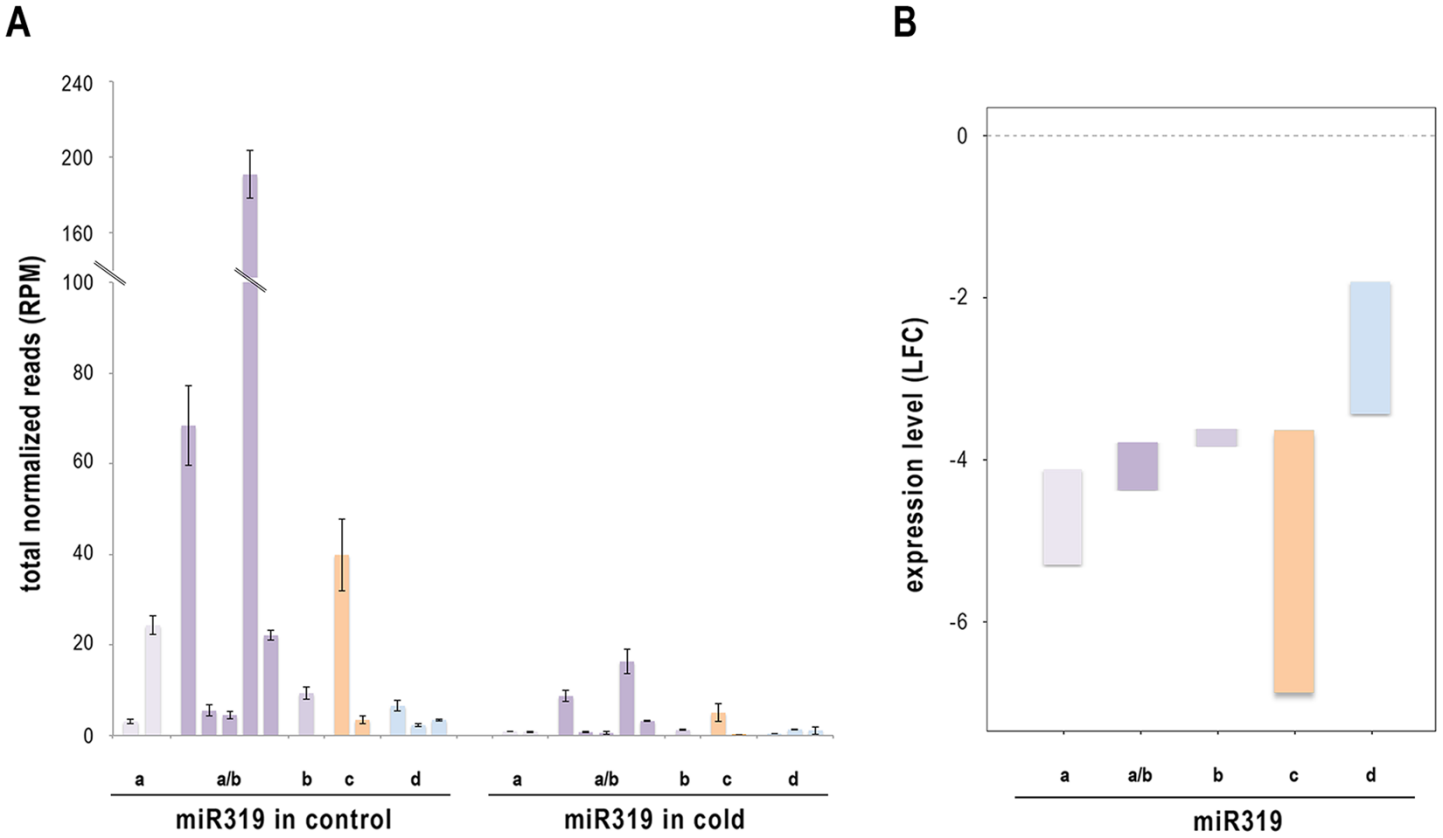
miR319 accumulates differentially in cold-exposed melon plants. (**A**) Graphic representation of the levels of the miR319 family members in melon plants exposed to cold treatments. The accumulation of miRNA in the analyzed samples is shown as the means of the total normalized reads (expressed in reads per million). Error bars show the confidence interval of the difference between means. (**B**) Relative expression levels (respect to the control) of the miR319 family members expressed as the Log fold change estimated by the statistical testing method edge-R. The boxes represent the range of expression values for the different sequences associated to each member precursor. More details are provided in Table S1.

Thirteen sRNAs (ranging from 20 to 22 nt) derived from the four miR319 precursors (a, b, c and d) described in melon (miRBase dataset) were recovered from our dataset and identified as cme-miR319 family members (Table S1). All of the miR319-related sequences analyzed (except for a miR319d-related sRNA) showed significantly reduced expression associated with cold-induced stress (Fig. 1A). The 21-nt sRNA corresponding to canonical melon miR319c exhibited higher downregulation (LFC −6.702) in plants exposed to low temperature conditions (Fig. 1B and Table S1).

### Reduced miR319c levels in cold-exposed plants are associated with higher pri-mRNA accumulation

**Figure 2:**
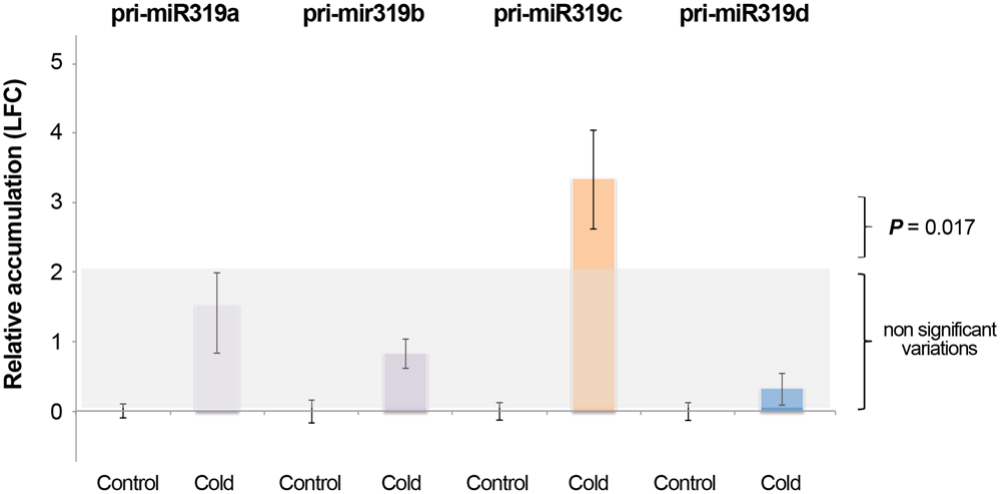
miR319c levels are not related to pri-miR319c accumulation. Relative accumulation of miR319 precursors in melon plants with respect to untreated controls exposed to a low temperature as estimated by qRT-PCR. The values on the Y-axis represent the mean of the expression (in Log fold change) for each one of the pri-miRNAs. Relative RNA expression was determined by the comparative ΔΔCT method and normalized to the geometric mean the expression of Profilin and ADP-ribosylation factor-like expression used reference controls. Error bars show the confidence interval of the difference between replicates. Control and cold-exposed plants were time-synchronized. The *P* value was estimated by paired t-Test analysis.

To determine whether the decrease in miR319 levels observed in cold-treated plants are associated with lower pri-miR319 accumulation, we quantified the levels of the miR319 precursor in cold-exposed and control plants via qRT-PCR. As shown in Fig. 2, a significant increase in pri-miR319c accumulation was observed in melon plants growing under this stress condition. By contrast, cold-exposed plants exhibited a significant decrease in miR319c accumulation together with a significant increase in pri-miR319c levels, supporting that the downregulation of miR319c accumulation is not related to lower transcriptional activity and/or the stability of its precursor.

### An alternative cold-responsive miRNA arises from the miR319c precursor

Once it was established that the downregulation of miR319c in cold-exposed plants was independent of the pri-miR319c levels, we attempted to analyze whether alterations in the sequential loop-to-base processing of its precursor^4,13,15^ might be responsible for this phenomenon. Small RNAs ranging from 20 to 25 nt recovered from the control and cold-exposed plant datasets were plotted by pairwise alignment (allowing only exact matching) against the four pri-miR319 sequences.

**Figure 3:**
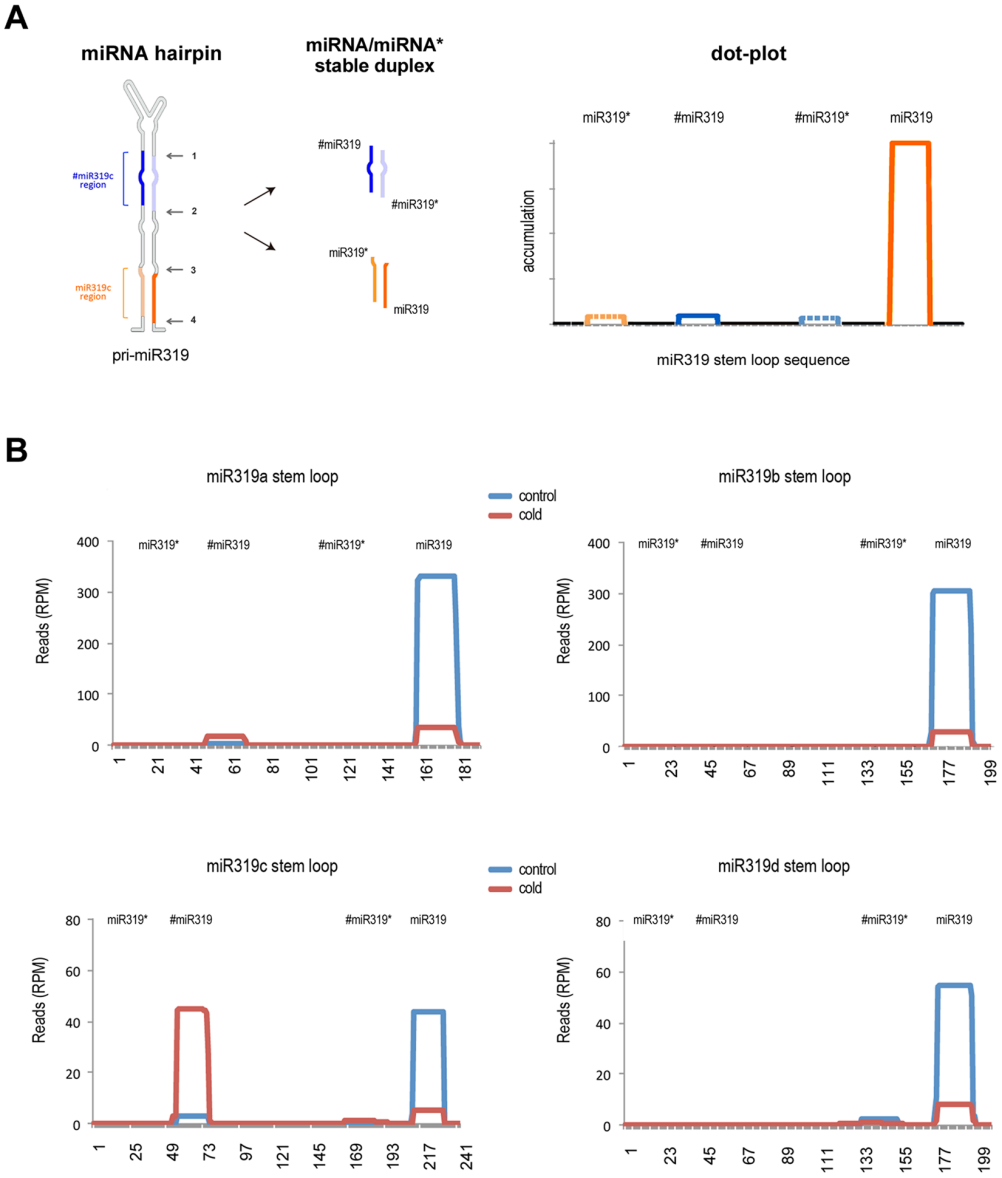
Alternative pri-miR319c processing is observed in cold-exposed melon plants. (**A**) Graphic representation of the predicted plot derived from conventional processing of the miR319 precursor. According the proposed loop-to-base processing^15^, only plots representing sequences derived from the stable miR319/miR319* duplex are expected. (**B**) The sRNAs (ranging 20 nt to 25 nt) recovered from the cold-exposed (red line) and control (blue line) melon plants were plotted (allowing only exact matching) onto the four pri-miR319 sequences described in melon. The values on the Y-axis represent the mean of the total reads in each library (normalized in reads per million). The nucleotide positions of the diverse pri-miR319 sequences analyzed are represented on the X-axis.

The sRNA distribution patterns expected from conventional precursor processing should be a prominent peak corresponding to the mature miR319 sequence and an occasionally reduced number of reads derived from the complementary miRNA* plotted on the opposite region in the hairpin (Fig. 3A). However, when small RNA reads recovered from cold-exposed and control plants were plotted onto different precursor sequences for the miR319 family, we observed that while the precursors of miR319b and d showed the expected distribution pattern, an extra peak appeared for miR319a and c, with the latter being particularly prominent and showing accumulation levels similar to that of miR319c (Fig. 3B). The two sRNA sequences corresponding to this extra peak were 20- and 21-nt long and were located in the 5′-arm of the hairpin, in contrast to mature miR319c, which arises from the 3′-arm (Table S2).

A detailed study of the sRNA sequences observed at the extra peak in the miR319c precursor showed that they corresponded to an additional miRNA (#miR319c) that was liberated as a consequence of non-canonical processing (Figs 3A and S2-upper panel). This multiple pri-miR319 processing has been precisely described in arabidopsis; however, in this species, the alternative miRNA/miRNA* duplex is unstable and is poorly recovered from the analyzed plants^15^.

Upon establishing the cold-dependent expression of #miR319c, we attempted to determine whether this alternative miRNA had any biological activity. Potential miR319c targets were predicted using psRNATarget^31^. Three melon transcripts (Phospho-2-dehydro-3-deoxyheptonate aldolase 2, 3-Ketoacyl-CoA synthase 20-like and the uncharacterized transcript LOC103494679), predicted as potential #miR319c targets, with expectation values ≤3.5 were selected to be analyzed by 5′-RLMN-RACE and qRT-PCR (Figure S3A). We were incapables to detect by 5′-RLMN-RACE transcript remnants with cleavage positions compatibles with AGO/miR319-processing (Figure S3B). In addition, the expected lower accumulation of predicted targets in cold exposed plants (overexpressing #miR319c) was not observed for any of the three predicted targets estimated by qRT-PCR. Altogether, our results indicate that there is no evidence to support any biological activity (referred to target cleavage) of this miRNA, at least regarding to this experimental approach.

### Alternative precursor processing is associated with the differential accumulation of miR319c and #miR319c

**Figure 4:**
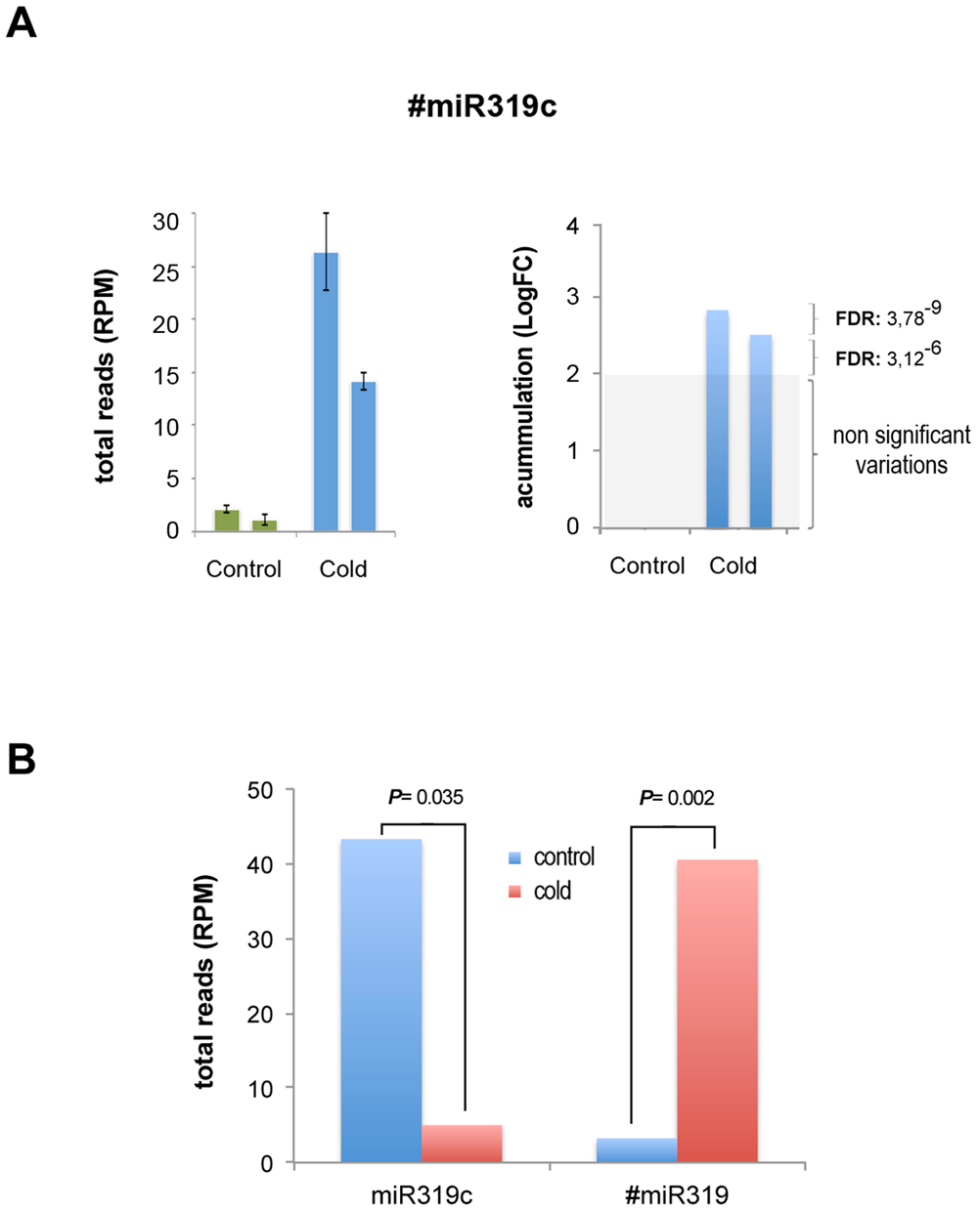
Accumulation of cold responsive #miR319c and miR319c is negatively correlated. (**A**) Graphic representation of the levels of the #miR319c sequences in melon plants exposed to cold treatment. The accumulation of miRNAs in the analyzed samples is shown as the mean of the total sequenced sRNAs (normalized in reads per million) (left) and the relative Log fold change accumulation (right) estimated by edge-R analysis. Error bars show the confidence interval of the difference between means. The double bar indicates the values of both miRNA-related sequences (more details in Table S2). (**B**) Histogram representing the sum of the means for the miR319c- (20 and 21nt length) and #miR319c (20 and 21nt length)-related sequences recovered from cold-exposed (red bars) and control (blue bars) plants. The *P* values were estimated by paired t-Test analysis.

When the accumulation of the sequences related to the extra peak detected in the miR319c precursor was analyzed, we observed that both #miR319c sequences showed significantly increased expression that was specifically associated with cold-induced stress (Figs 4A and S4), indicating the existence of an inverse correlation between the read numbers recovered for miR319c and extra miRNA (Fig. 4B). In both control and cold-exposed samples, a high level of one miRNA type (miR319c or extra-#miRNA) necessarily implies a significant reduction in the accumulation of the other. The alternative #miRNA was also observed when sRNAs were plotted onto the miR319a precursor. However, in this case, the differential accumulation of #miRNA in cold-exposed plants was not significant (Figure S5). A similar analysis performed against precursors for the rest of the cold-responsive melon miRNAs (Figure S6) showed that the change in precursor processing associated with low temperature was a phenomenon restricted to pri-miR319c.

Our results support the idea that the significant decrease in miR319c observed in melon plants exposed to low temperature could be a consequence of changes in its precursor processing, favoring the accumulation of miR#319c instead of canonical miR319c.

### Partial cleavage and misprocessing of its precursor modulates miR319c accumulation

**Figure 5:**
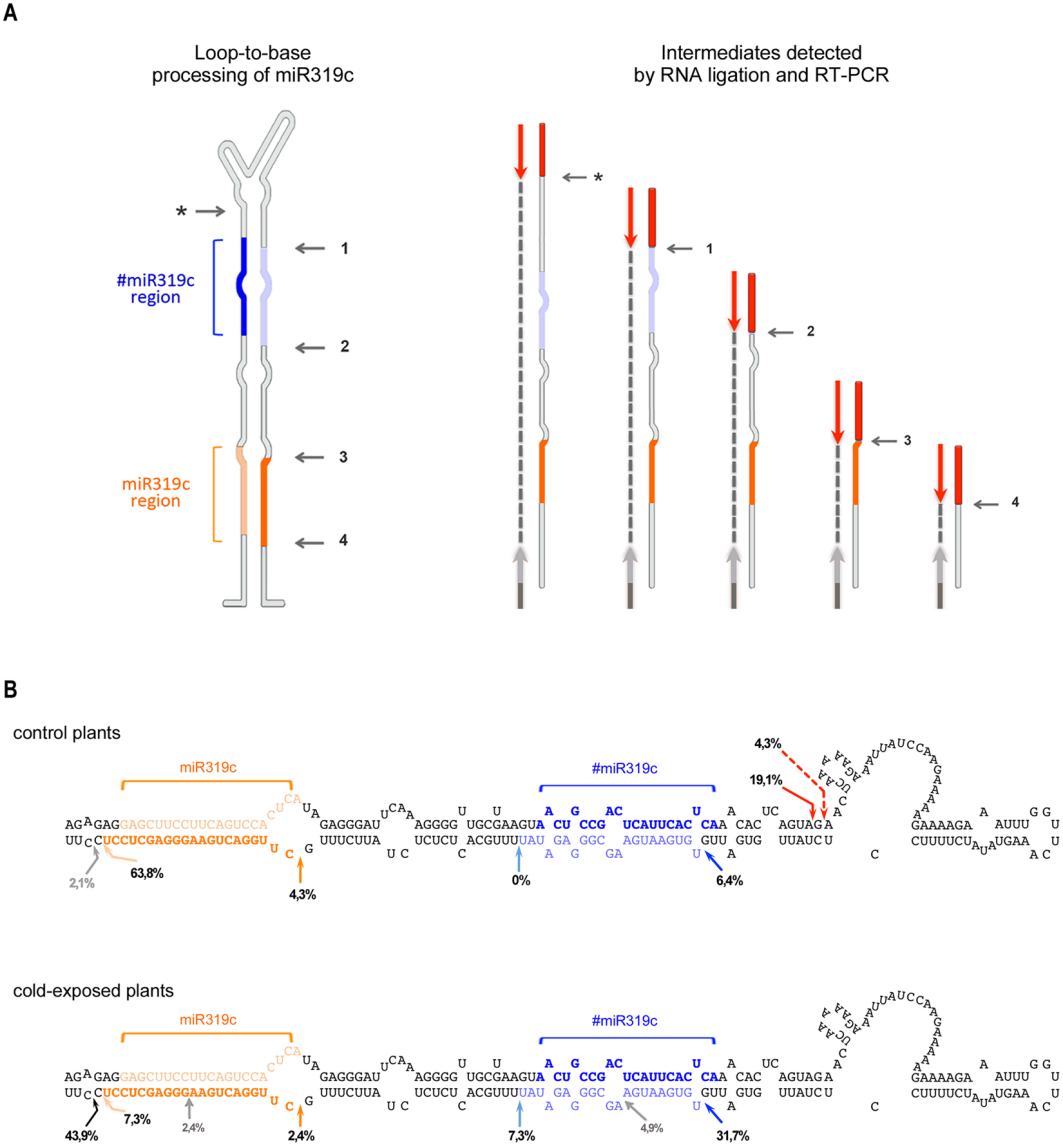
Processing of the miR319c precursor in melon, as estimated by 5′RACE. (**A**) Scheme illustrating the method used to identify processed precursor intermediates and the expected fragments. An (*) indicates the partial cut in the 5′-arm. (1 to 4) Represent the four cleavage reactions expected for the canonical loop-to-base-processing of the miR319c precursor. The red bars represent the 5′-RNA-adaptor. Arrows indicate the position of the oligos used in the last PCR amplification. (**B)** Schemes showing the predicted secondary structure (estimated from sequence > METC022194) and abundance (in percentage) of the cleavage sites detected among processed miR319c precursor recovered from control (upper panel) and cold-exposed (lower panel) plants. Red arrows (filled and doted) show partial processing points in the 5′-arm. Blue and magenta arrows indicate processing points 1 and 2. The processing points related to cuts 3 and 4 are marked with orange arrows. Black arrows indicate the position of the unexpected dicing event involved in the release of unstable miR319c. Gray arrows show the less abundant and unspecific cleavage site.

To explore whether precursor processing is associated with the unbalance in #miR319c and miR319c accumulation, we analyzed pri-miR319c intermediates in cold-exposed and control plants using a modified 5′-rapid amplification of cDNA ends (RACE) method. Due to the relative position of the oligos, this method allows for the detection of the four cleavage positions in the loop-to-base processing of miR319c in the 3′-arm (Fig. 5A). Sequence data showed that the major cleavage sites detected in the processed intermediates amplified from non-treated melon plants were expected for canonical loop-to-base-processing (Fig. 5B). Approximately 68% of the analyzed reads matched the exact cuts at sites 3 (4,3%) and 4 (63,8%), which are involved in the release of the canonical miR319c/miR319c* duplex, while a minor fraction (6,4%) of the detected sequences corresponded to precursor fragments originating from a cut at site 1, which is responsible for #miR319c/#miR319c* biogenesis. Unexpectedly, a considerable proportion (19,1%) of the recovered reads matched to one specific position in the 5′-arm of pri-miR319c (marked with an asterisk in Fig. 5A and with red arrows in Fig. 5B). According to the experimental design of the RACE assay, the cuts detected in the 5′-arm of pri-miRNA implicate the existence of partially cleaved intermediates^7^. A lower abundance cut (4,3%) was also detected adjacent to the preponderant cut position in the 5′-arm of miR319c (dotted arrow). The partial cleavage in the 5′-arm was not detected when processed intermediates that arose from cold-exposed plants were evaluated (Fig. 5B - lower). We observed that 39% of the obtained reads matched the exact cuts at sites 1 (31,7%) and 2 (7,3%), which are involved in the release of the #miR319c/#miR319c* duplex. By contrast, only 9,7% of the sequenced fragments corresponded to canonical products from cut sites 3 (2,4%) and 4 (7,3%). The rest of the sequenced products (43,8%) corresponded to precursors processed at a non-canonical position adjacent to cut site 4 (marked with a black arrow in Fig. 5B - lower). Although this unexpected dicing position was predominantly observed during pri-miR319 processing in cold-exposed plants, the 22-nt mature miR319c that arose from this dicing event was not recovered from sRNA libraries derived from melon plants exposed to this environmental condition.

To provide additional evidence supporting the existence of the partial cleavage observed among the processed precursor recovered from control plants, we designed a more specific 5′-RACE strategy (Figure S7A). The results showed that remnants of the partially processed miR319c precursor were only found in intermediates recovered from control plants (Figure S7B). Equivalent partial cuts in the 5′-arm were also not detected when miR319a precursor processing was analyzed (Figure S8), supporting the idea that this unusual event might be restricted to pri-miR319c.

### The cold-induced decrease of miR319c correlates with the increased accumulation of well-established members in the low temperature signaling pathway

In an attempt to clarify the relationship between the low temperature-induced decrease in miR319c and cold acclimation, we analyzed its potential regulatory effects in melon plants. It is well-established that TEOSINTE BRANCHED1/CYCLOIDEA/PROLIFERATIN CELL FACTOR (TCP) mRNA is a highly conserved target of miR319 family members in diverse vegetal species^20,32^. In melon, although both the TCP2 and TCP4 transcripts were predicted to be recognized by miR319c according to psRNA target estimations, TCP2 appeared to be the primary target for miR319c activity (Figure S9).

**Figure 6:**
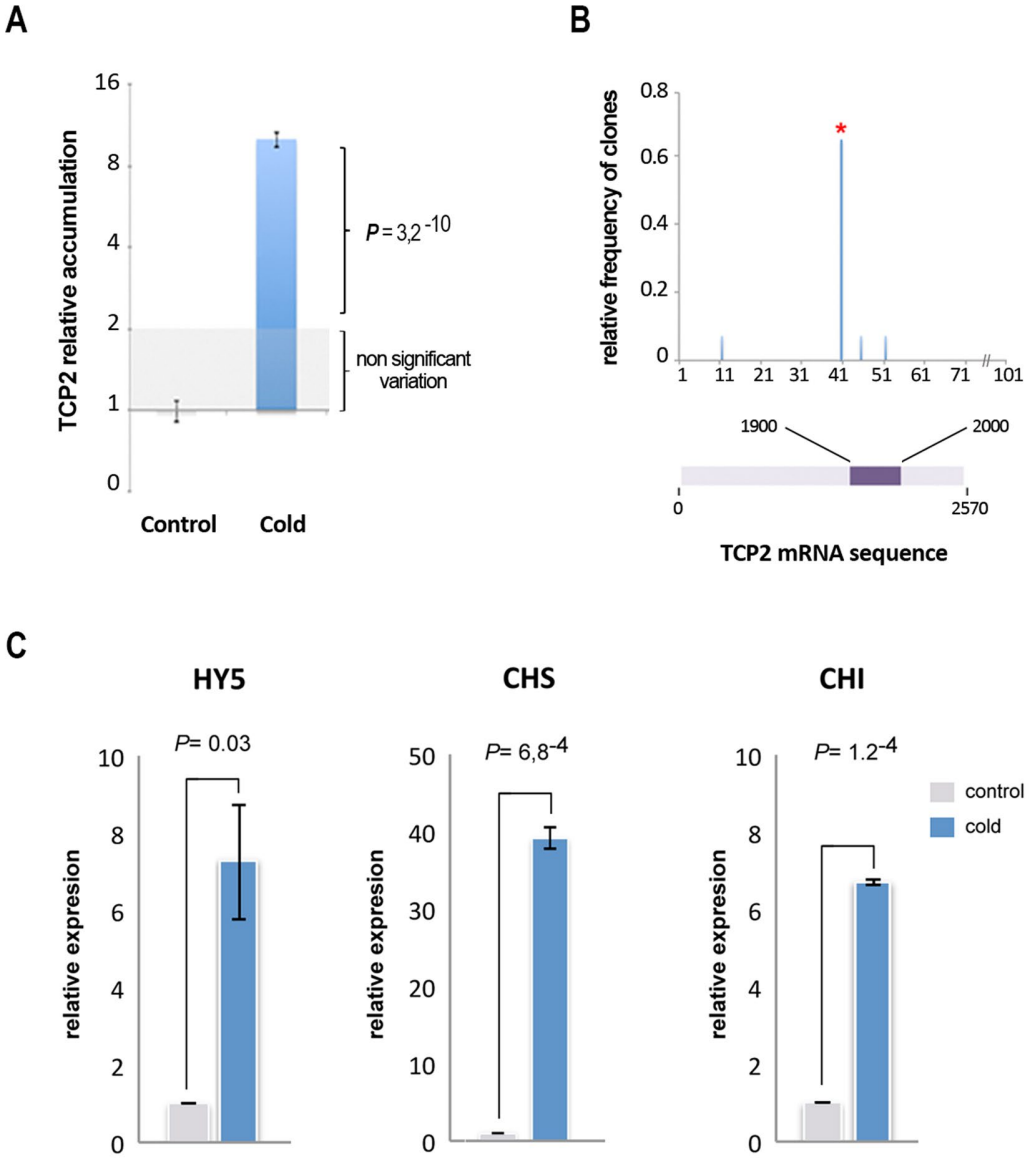
Cold-induced decrease in miR319c correlates with increased accumulation of well-established members of the low temperature signaling pathway. (**A**) Histogram showing the means of the relative accumulation of TCP2 mRNA in melon plants exposed to cold as estimated by qRT-PCR-. Error bars show the confidence interval of the difference between means. (**B**) Representation of the cleaved TCP2 transcript detected by the 5′-RLM-RACE assay. The X-axis indicates the nucleotide position in a selected region (dark magenta box in graphics) of the TCP2 transcripts. The Y-axis shows the relative frequency of clones sequenced showing cleavage in this position. The red asterisk indicates the expected position for TCP2 cleavage mediated by miR319. (**C)** Relative accumulation with respect to the untreated control for the *ELONGATED HIPOCOTYL 5* (HY5), *CHALCONE SYNTHASE* (CHS) and *CHALCONE ISOMERASE (CHI)* homologous transcripts in melon plants exposed to low temperature for 11 days as estimated by qRT-PCR. Error bars show the confidence interval of the difference between means. The *P* value was estimated by paired t-Test analysis.

Quantitative real time-PCR (qRT-PCR) assays revealed that TCP2 accumulation was significantly increased in cold-treated plants, indicating that the stress-induced decreased in miR319c correlated with the expected functional regulation of its predicted mRNA target (Fig. 6A). This observation was reinforced by the majoritarian detection (9 out of 14 analyzed clones, 64%) of AGO/miR319-sliced TCP2-mRNA remnants with cleavage positions between nucleotides 10 and 12 relative to the 5′-end of the miRNA, estimated by RLM-5′-RACE (Fig. 6B).

It has recently been reported that TCP2 positively regulates the accumulation of *ELONGATED HIPOCOTYL 5* (HY5) in *Arabidopsis* plants^33^. HY5 is a bZIP transcription factor that in addition to its central role in photomorphogenesis^34,35^ also acts as a positive regulator of cold acclimation in *Arabidopsis* ^36–38^ by activating key components in the anthocyanin biosynthesis pathway^39^. To evaluate whether the increased TCP2 levels observed in cold-exposed melon plants (Fig. 1B) could be associated with this response mechanism to low temperatures described in arabidopsis, we first determined the levels of the melon homologs of arabidopsis HY5. As shown (Fig. 6C, left panel), accumulation of the predicted HY5 transcript was significantly increased in cold-exposed melon plants, suggesting that miR319c may be involved in the regulation of HY5 via TCP2 in melon.

Next, we analyzed the accumulation of transcripts predicted to encode melon homologs of critical enzymes in anthocyanin biosynthesis, such as *CHALCONE SYNTHASE* (CHS) and *CHALCONE ISOMERASE (CHI)* ^40^. The qRT-PCR results showed that the expression of both (CHS and CHI) melon transcripts was significantly increased in plants growing under low temperature (Fig. 6C, central and right panels), supporting the existence of a possible regulatory link between the cold-induced decrease in miR319c and low temperature signaling pathway mediated by HY5 in melon.

## Discussion

miRNAs play a fundamental role in controlling diverse aspects of plant development and plant-environment interactions^2,41^. In the last few years, numerous studies in various animals have shown that there is a strong correlation between the stress-response and differential accumulation of certain miRNAs^9,42,43^. Although our understanding of the link between plant-miRNA expression and adverse environmental conditions has been greatly improved, many questions remain to be fully deciphered. Elucidation of the mechanism regulating the processing of specific pri-miRNAs under stress conditions is an interesting challenge^9^.

To address this issue, we analyzed miR319 accumulation in melon plants exposed to low temperature conditions. Consistent with results observed in other plants, such as rice^22^, Arabidopsis^24^ and sugarcane^23^, our results indicate that melon members of the miR319 family are stress-responsive miRNAs. Although miR319a, miR319b and miR319d were also downregulated in plants exposed to cold, miR319c showed a greater decrease, suggesting that it may be predominantly regulated under this stress situation.

**Figure 7:**
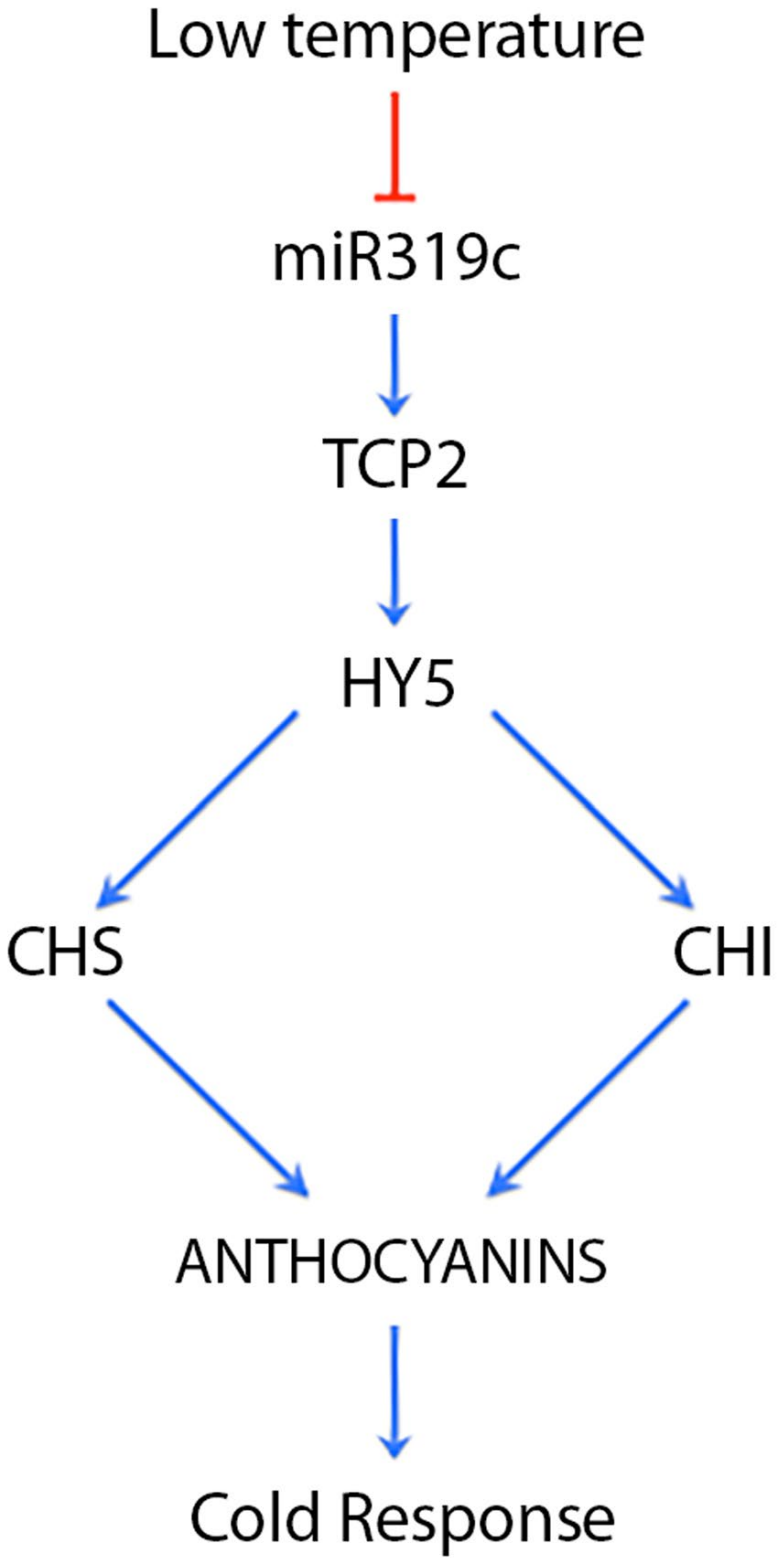
Proposed model for miR319c function in response to cold in melon. Low temperature induced a decrease of miR319c that promoted a TCP2-mediated increase in HY5 that transcriptionally modulated the accumulation of key components in the anthocyanin biosynthesis pathway (CHS and CHI), which is involved in cold response.

Furthermore, the observation that the decrease in miR319c may be associated with the transcriptional regulation of key components in the anthocyanin biosynthesis pathways involved in low temperature signaling via negative regulation of HY5 mediated by TCP2^37^ suggests a functional role for this melon miRNA in the cold response (Fig. 7). Further studies are necessary to reliably determine the existence of this regulatory link and establish whether miR319c is a unique regulatory factor that modulates the TCP2/HY5-mediated response^33^ during the acclimation process in melon plants. Additionally, the observation that the direction of differential miR319 expression varied among analyzed plants, decreased in melon and rice^22^ and increased in arabidopsis^24^ and sugarcane^23^, indicates that the miR319-mediated response to cold is regulated in a species-specific manner, which is consistent with observations of other miRNAs affected by stress^9^.

The evidence accumulated to date indicates that there is a positive correlation between the accumulation of a particular miRNA and its corresponding precursor. However, we observed that cold-stressed melon plants exhibited a significant decrease in miR319c accumulation together with a significant increase in pri-miR319c levels. This result indicates that miR319c accumulation is independent of pri-miRNA transcription and/or stability, suggesting the possibility that the decrease in miR319c observed in melon plants exposed to low temperatures could be a consequence of alterations in its precursor processing. To evaluate this possibility, we analyzed the distribution of sRNAs derived from the pri-miR319c sequence and recovered them from both control and cold-exposed plants by pairwise alignment. This study revealed that the decrease in miR319c accumulation was strictly correlated with a significant increase in the accumulation of an alternative miRNA derived from the miR319c precursor. This non-canonical miRNA, named #miR319c, was identified as the melon homolog of the miRNA/miRNA* duplex derived from the first DCL1-mediated cuts performed during miR319 biogenesis in *Arabidopsis* ^15^.

Our data showed that the accumulation of miR319c in melon was strictly dependent on the stability of #miR319c, which arises from the upper region of its precursor in response to low temperature conditions. These observations are consistent with a previous work performed in arabidopsis showing that the accumulation of the alternative miRNA derived from the miR319 precursor compromises the biogenesis of mature canonical miR319 in mutant plants^15^ and during plant development^17^.

The evidence accumulated so far supports the idea that four DCL1-mediated cleavages are necessary for the miR319/miR319* duplex to be finally released during loop-to-base processing of miR319^13,15^. Our analysis of the processed precursors in untreated plants demonstrates that although melon miR319c biogenesis includes multiple dicing events, which are conserved from the moss *Physcomitrella patens* to arabidopsis^13,15,16^, an additional partial cut in the 5′-arm of the upper stem of its precursor may also be involved in pri-miR319c processing.

Identification of these intermediates by 5′-RACE support the notion that they are likely the result of miR319c precursor misprocessing by RNase III enzymes, resembling, at least in part, the DCL1-mediated misprocessing events described during miR171a/b, miR393b and miR166b biogenesis in Arabidopsis^7^. Interestingly, this partial cleavage site was not found when processed precursors recovered from cold-exposed plants were analyzed, showing that the normal loop-to-base-processing of melon pri-miR319c is impaired in plants that grow under this environmental condition. Considering that cold-exposed plants (which lack the partial cut during precursor processing) exhibit a significant decrease in mature miR319c levels, it is reasonable to assume that this additional dicing event might favor the accumulation of the canonical miR319c/miR319c* duplex in melon plants.

The additional partial cut was also not detected in processed miR319a precursors recovered from control plants. Interestingly, when we investigated the secondary structure predicted for pri-miR319c and pri-miR319a at a control temperature (28 °C) and for pri-miR319c under cold treatment temperature (20 °C), we observed that both precursors that lacked the additional partial cleavage exhibited a more stable hairpin structure (Figure S10). This result is consistent with the notion that the unstructured RNA domains that are predicted to exist in the miR319c precursor (in plants maintained under standard temperature for melon development, 28–30 °C) might be associated with the partial cut observed during conventional miR319c biosynthesis. In this sense, a previous work has already shown the existence of competing RNA domains in the same precursor that can be recognized by the miRNA processing machinery^44^.

The mechanistic causes that promote the decrease in miR319c in cold-exposed melon plants remain to be established; however, the observation that the dicing at position 4 (involved in the release of the canonical miR319c/miR319c* duplex) is strongly altered under stress conditions allows us to establish a link between the absence of the additional partial cut and alterations in the processing accuracy of the miR319c precursor. miR319c (22-nt), which is expected to be released as a consequence of an altered cut at the position 4, is not detected in cold-exposed plants, suggesting that this non-canonical miR319c/miR319c* duplex is highly unstable.

**Figure 8:**
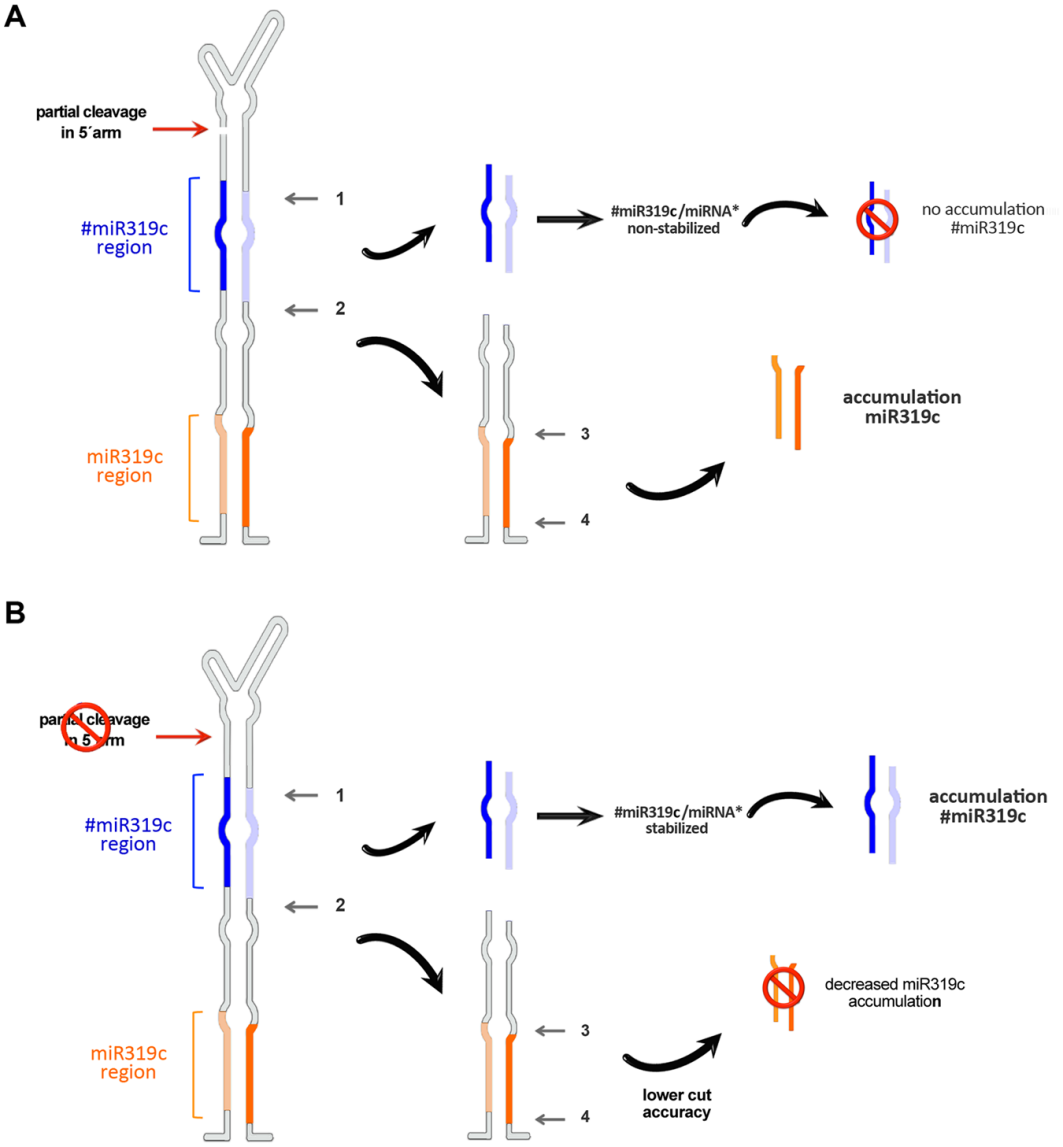
Proposed model for the processing of miR319c in melon. (**A**) The miR319c precursor is first partially cleaved at the 5′-arm. DCL1 then continues to cut the precursor four more times in a loop-to-base direction until mature miR319c is finally released. The alternative #miR319c/miRNA* duplex is unstable under these processing conditions. (**B**) In cold-exposed plants, the first partial cleavage is impaired, promoting lower accuracy in the next four DCL1-mediated cuts (mainly cut 4). As a consequence of this altered precursor processing, stable miR319c/miRNA* is inefficiently released and/or accumulated. By contrast, accumulation of alternative #miR319c is increased.

Altogether, our results are consistent with the notion that the processing of the miR319c precursor might begin with a partial cleavage in the upper stem of the hairpin in melon plants. Homologous melon DCL1 then continues to cut the precursor four more times in a loop-to-base direction until mature miR319c is finally released (Fig. 8A). Consistent with observations in arabidopsis, this multiple pri-miRNA dicing might lead to lower alternative #miR319c accumulation. Under low temperature conditions and due to unknown causes, which may be related to the RNA secondary structure, the first partial dicing is impaired. The lack of this first processing event is associated with a lower efficiency and accuracy for the two DCL1-mediated cuts (mainly cut 4), which are involved in miR319c release, promoting a significant decrease in canonical miR319c accumulation. Moreover, and as consequence of this altered biogenesis pathway, the processing and accumulation of alternative #miR319c is strongly increased (Fig. 8B).

Similar intermediates produced by partial cleavage have also been detected in plant^7^ and animal^45,46^ cells; however, the functional basis and regulatory implications of this unusual processing remain to be elucidated^7^. The observation that the lack of this partially processed intermediate impairs pri-miRNA biogenesis provides new insights into the versatility of plant miRNA processing and the mechanisms regulating them in response to environmental conditions.

## Methods

### Plant material, growth conditions and stress treatments

Melon seeds from cv. *Piel de Sapo* were germinated in Petri dishes at 37 °C/48 h darkness and 25 °C/24 h (16/8 light/darkness). Melon seedlings were sown in pots and maintained for 10 days under controlled conditions (28 °C/16 h light and 20 °C/8 h darkness) in a growing chamber. Only homogeneously developed plants were selected for stress treatment. Next, the melon plants were exposed to low temperature (20 °C/16 h light and 14 °C/8 h darkness) treatment for 11 days. Three replicates were performed for cold treatment and four for controls. Each analyzed sample is a pool of three plants. The plants were irrigated alternatively (water and Hoagland solution) by inundation (1500 ml/48 h). Control plants were maintained at 28 °C/16 h light and 20 °C/8 h darkness.

### RNA extraction and sRNAs purification

Total RNA was extracted from pooled leaves (~0.1 g) recovered from treated and control melon plants using the TRI reagent (SIGMA, St. Louis, MO, USA) according to the manufacturer’s instructions. Three biological replicates were performed for cold treatment and four for the controls. Each biological replicate corresponds to a pool of three treated plants. The low-molecular weight RNA (<200 nt) fraction was enriched from total RNA using TOTAL-miRNA (miRNA isolation Kit, REAL) according to the manufacturer’s instructions.

### sRNAs sequencing

Production and sequencing of the libraries were performed by the company SISTEMAS GENOMICOS SL (https://www.sistemasgenomicos.com). cDNA libraries (three replicates for stress treatment and four for controls) were obtained following Illumina′s recommendations. Briefly, 3′- and 5-adaptors were sequentially ligated to the RNA prior to reverse transcription and cDNA generation. The cDNA was enriched with PCR to create the indexed double-stranded cDNA library. Size selection was performed using a 6% polyacrylamide gel. The quantity of the libraries was determined by real-time PCR in a LightCycler 480 (Roche). Prior to cluster generation in cbot (ILLUMINA), an equimolar pooling of the libraries was performed. The pool of the cDNA libraries was sequenced by paired-end sequencing (100 × 1) in a HiSeq2000 (ILLUMINA). Adaptors and low quality reads were trimmed by using the Cutadapt tool (v. 1.10) in Python^47^. The correlation exhibited by the miRNA expression profiles between treated and control samples, was established by a Principal Component Analysis (PCA) and *Pearson* correlation. PCA was performed with an R package as previously described^48^. The sequencing data were deposited in the genomic repository SRA of the NCBI (BioProject ID PRJNA491809; http://www.ncbi.nlm.nih.gov/bioproject/491809).

### Bioinformatics analysis of miR319 expression

The statistical testing method edegR-Bioconductor v3.14^49^ (https://bioconductor.org/packages/release/bioc/html/edgeR.html) was used to evaluate the differential expression of miR319 in melon plants under stress conditions. The sRNAs identified as differentially expressed (log2-fold change (log2FC) ≥2.0 or ≤−2.0 and an *FDR* value ≤ 0.05) were aligned to the miRNA database miRBase v21.0 using blastall v2.2.17^50^. Only the sRNAs that were fully homologous to previously described members of the cme-miR319 family (http://www.mirbase.org) were analyzed in this work.

### sRNA alignment

Small RNAs ranging from 20 to 25 nt recovered from the control and cold-exposed plant dataset were aligned against the four pri-miR319 sequences (recovered from the miRBase dataset) using Bowtie -v1.1.19-^51^ with the number of mismatches allowed set to zero. Next, we used SAMtools -v0.1.19-^52^ to generate the alignment files. The sRNAs aligned against pri-miR319 were normalized to reads per million using BEDTools suite -v2.25.0-^53^.

### Real-time quantitative PCR assays

Total RNAs (1.5 μg) extracted as described above from control or treated plants were subjected to DNase treatment (EN0525, Thermo Scientific™) followed by reverse transcription using the RevertAid First Strand cDNA Synthesis Kit (Thermo Scientific™) according to the manufacturer’s instructions for use with oligo dT. To assess the reaction specificity, cDNAs were amplified by conventional end-point RT-PCR using specific primers (Table S3) and the PCR products were sequenced. Quantification of miRNA-targets was performed by quantitative real-time RT-PCR. The 20 μl PCR reaction contained 1X Pyro Taq EvaGreen qPCR Mix (Cultek), 0.6 μM of each primer and varying amounts of RT products depending on the target abundance. All measurements were performed in triplicate on an ABI 7500 Fast-Real Time qPCR instrument (Applied Biosystems) using a standard protocol, including a dissociation step (ramping from 60 to 95 °C) to monitor the melting curve of the amplification products. The PCR amplification efficiency was derived from a standard curve generated by four 10-fold serial dilution points of cDNA mixed from all the samples. Relative RNA expression was determined by using the comparative ΔΔCT method^54^ and normalized to the geometric mean of Profilin (NM_001297545.1) and ADP-ribosylation factor-like (XM_008463181.2) expression, as reference controls. The statistical significance of the observed differences was evaluated by the paired t-Test.

### 5′-RLM-RACE

Identification of the cleaved transcripts was performed by 5′-RLM-RACE^55^ according to the GeneRacer kit guide (Invitrogen, Carlsbad, CA) with minor modifications. Briefly, a 5′-RNA adaptor was directly ligated to the 3′-end of the cleavage products with a ligation-competent 5′-monophosphate in a reaction containing 100 ng of polyA(+) RNA purified from total RNA using an Oligotex mRNA Midi Kit (Qiagen). The ligated RNAs were then reverse-transcribed with the GeneRacer oligo dT primer (as described above). These cDNAs were amplified through universal PCR with 5′- and 3′- GeneRacer primers. This product served as a template for the specific amplification of the cleaved transcripts by two consecutive PCR reactions with the 5′-GeneRacer primer and two RNA-specific nested primers (Table S3). The PCR products were cloned and sequenced, allowing for the determination of the RNA cleavage sites.

## Electronic supplementary material

Supplementary Information
