# Author Correction: Alternative processing of its precursor is related to miR319 decreasing in melon plants exposed to cold

**DOI:** 10.1038/s41598-022-22793-x

**Published:** 2022-11-02

**Authors:** Antonio Bustamante, Maria Carmen Marques, Alejandro Sanz-Carbonell, Jose Miguel Mulet, Gustavo Gomez

**Affiliations:** 1 Institute for Integrative Systems Biology (I2SysBio), Consejo Superior de Investigaciones Científicas-Universitat de Valencia (CSIC-UV), Parc Cientific UV, Catedratico Agustin Escardino 9, 46980 Paterna, Spain; 2 Instituto de Biología Molecular Y Celular de Plantas (IBMCP), Consejo Superior de Investigaciones Científicas (CSIC)-Universitat Politecnica de Valencia (UPV), CPI, Edificio 8 E, Av. de los Naranjos S/N, 46022 Valencia, Spain; 3 Instituto Nacional de Investigaciones Agropecuarias (INIAP), Estación Experimental Pichilingue, Km5 Vía Quevedo El Empalme, Mocache, Ecuador

Correction to: *Scientific Reports* 10.1038/s41598-018-34012-7, published online 19 October 2018

The Acknowledgements section in the original version of this Article is incomplete.

“The authors thank Drs G. Martinez for its critical reading of this manuscript and A. Monforte for providing melon seeds.”

should read:

“The authors thank Drs G. Martinez for its critical reading of this manuscript and A. Monforte for providing melon seeds. This work was supported by grant AGL2016–79825-R from the Spanish Ministry of Economy and Competitiveness (co-supported by FEDER). The funders had no role in the experiment design, data analysis, decision to publish, or preparation of the manuscript.”

