# Supplementary material for: Alternative processing of its precursor is related to miR319 decreasing in melon plants exposed to cold
Source: Sci Rep. 2018 Oct 19;8:15538. doi: 10.1038/s41598-018-34012-7 (PMC6195573; doi:10.1038/s41598-018-34012-7)
Supplement: Supplementary file 1 — Supplementary Information [file 41598_2018_34012_MOESM1_ESM.pdf]

Supplementary Information

Manuscript Title:
Alternative processing of its precursor is related to miR319 decreasing in melon plants exposed to cold

Authors
Antonio Bustamante, Maria Carmen Marques, Alejandro Sanz-Carbonell, Jose Miguel Mulet and Gustavo Gomez*

Detail of submitted information

Figures Supplementary S1 to S10
Tables supplementary Table S1 to Table S3

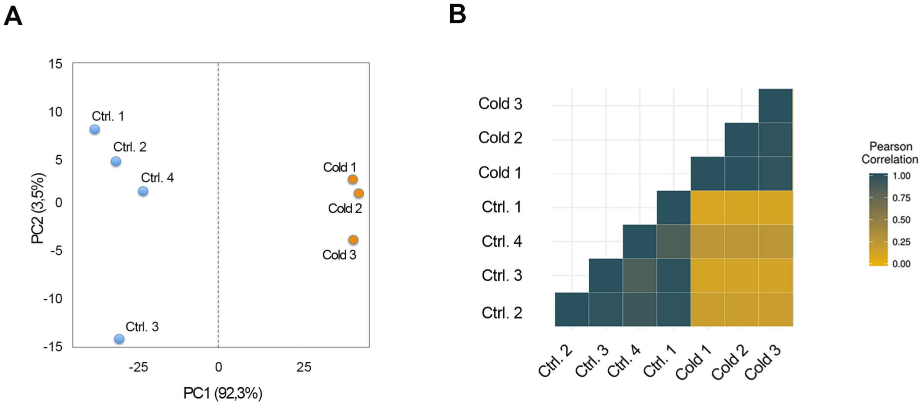

Figure S1: Analysis of the sRNA population recovered from analyzed melon libraries. A) Correlations between sRNA expression profiles (considering the different treatments and their biological replicates) were estimated by principal component analysis (PCA). The PCA plot shows that biological replicates of the cold treated samples are clustered together and clearly distinguished from control, attesting to the reproducibility of our assays. **B)** Heat map showing the pair-wise Pearson rank correlation between the sRNAs read of the analyzed libraries. As is showed in the figure, high correlation values (>0,88) were obtained between replicates. The color key represents the correlation coefficient range.

```
                              #miR319
pri-mir319a    AGGG---TTTAATTAGCTGCCGACTCATTCATTCAAATA
pri-mir319b    TCGGTT-GTTACTTAGCTGCTGACTCGTTGGCTCAATAA
pri-mir319c    GGGGTTGCTGAAGTAACTGCCGACTCATTCACTCAAACA
pri-mir319d    AAGGTC-GAAACGTTGCTGCTCATTCGTTAGTTCAATAG
               --**------*--*--****--*-**-**---****---
```

```
                              miR319
pri-mir319a    GCTTCCTGTACTTGGACTGAAGGGAGCTCCCTTTTCTTTC
pri-mir319b    GCTGTCACGTCTTGGACTGAAGGGAGCTCCCTCACCTTCC
pri-mir319c    TTATTCTTTGCTTGGACTGAAGGGAGCTCCTCCTTTTTAC
pri-mir319d    GTTGTCATGTATTGGACTGAAGGGAGCTCCTTCACTTTTA
               -----*-----*******************------**--
```

Figure S2: Conservation of miR319 and #miR319 in melon. The sequences of the four miR319 stem-loops (a, b, c and d) described in melon were aligned. Only the precursor region corresponding to #miR319 (orange box) and miR319c (blue box) mature forms are detailed. Conserved nucleotides are marked with asterisk.

A

Input	Target ID	Expect	Sample	Degradome sequences matching to target (%)								Target description
				< -74 nt	-74 / -36	-35 / -16	-15 / -1	0 / 14	15 / 34	35 / 74	> 75	
AACTGCCGACTCATTCACTCA	METC017726	2.0	Cold	0	0	0	0	0	0	0	100	*C. melo phospho-2-dehydro-3-deoxyheptonate aldolase 2, chloroplastic*
AACTGCCGACTCATTCACTCA	METC003436	3.5	Cold	0	0	0	0	0	40	60	0	*C. melo 3-ketoacyl-CoA synthase 20-like*
AACTGCCGACTCATTCACTCA	METC008902	3.5	Cold	100	0	0	0	0	0	0	0	*C. melo uncharacterized LOC103494679*

B

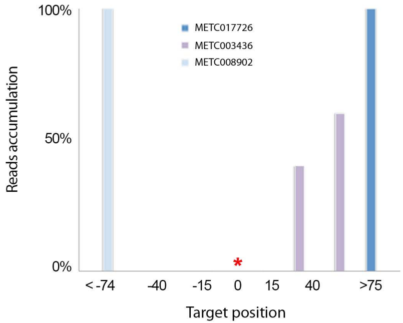

C

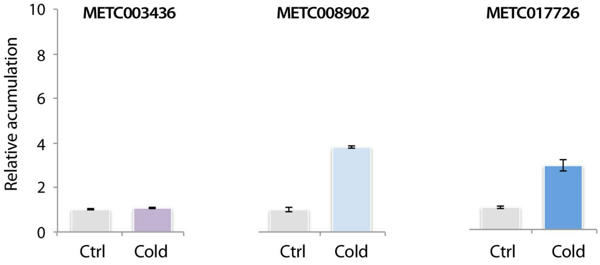

Figure S3: #miR319c no posses demonstrable biological activity. A, Detail of the predicted targets for #miR319c in melon (only transcripts with expectation values ≤3.5 were selected to be analyzed). **B,** Representation of cleaved transcripts detected by 5′ RLM-RACE assay. The X axis indicates the nucleotide position on selected region of the analyzed transcripts. The Y axis show the relative frequency of clones sequenced showing cleavage in this position (referred to 10 clones analyzed for each transcript). The red asterisk indicates the expected position for transcripts cleavage mediated by #miR319c. As be observed, no canonical #miR319c-cleaved products were obtained for the three potential targets analyzed. **C,** To additionally evaluate the biological function of #miR319c, predicted targets accumulation was estimated by qRT-PCR in both cold-treated (with increased #miR319c accumulation) and control plants. The obtained data indicate that the expected lower accumulation of predicted targets in cold exposed plants, was not observed for any of the three analyzed transcripts.

A

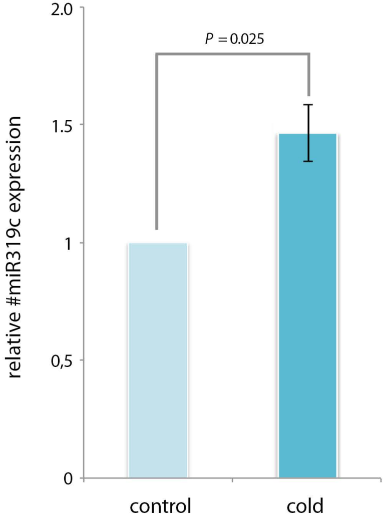

B

**miR159**	SEQUENCE	**TTTGGATTGAAGGGAGCTCTA**
	RT-miR159	GTTGGCTCTGGTGCAGGGTCCGAGGTATTCGCACCAGAGCCAACtagagc
	miR159-Fw	gcgccTTTGGATTGAAGGGAG

**miR#319c**	SEQUENCE	**AACTGCCGACTCATTCACTCA**
	RT-miR#319	GTTGGCTCTGGTGCAGGGTCCGAGGTATTCGCACCAGAGCCAACtgagtg
	miR#319-Fw	gcgcaAACTGCCGACTCATT

Figure S4: Validation of differential #miR319c accumulation. A) Relative accumulation with respect to untreated control, estimated by stem loop qRT-PCR, of #miR319c in melon plants exposed for 11 days to cold. Analyses were performed in triplicate using a standard protocol. The efficiency of PCR amplification was derived from a standard curve generated by four five-fold serial dilution points of cDNA mixed from the two samples. RNA expression was quantified by the comparative ΔCt method and normalized to the geometric mean of expression of miR159 as reference control. Statistical significance of the observed differences was evaluated by paired t-Test. **B)** Primers used are listed in Table.

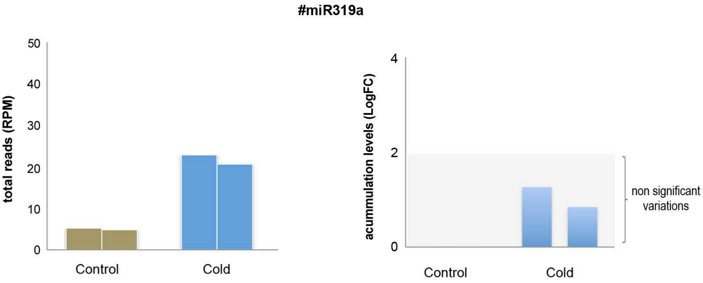

Figure S5: **#miR319a exhibit non-significant diferential acummulation in cold exposed plants.** Graphic representation of levels of #miR319a sequences in melon plants exposed to cold treatment and controls. The accumulation of miRNAs in the analyzed samples is showed as total normalized reads (left) and relative Log fold change accumulation (right) estimated by edge-R analysis.

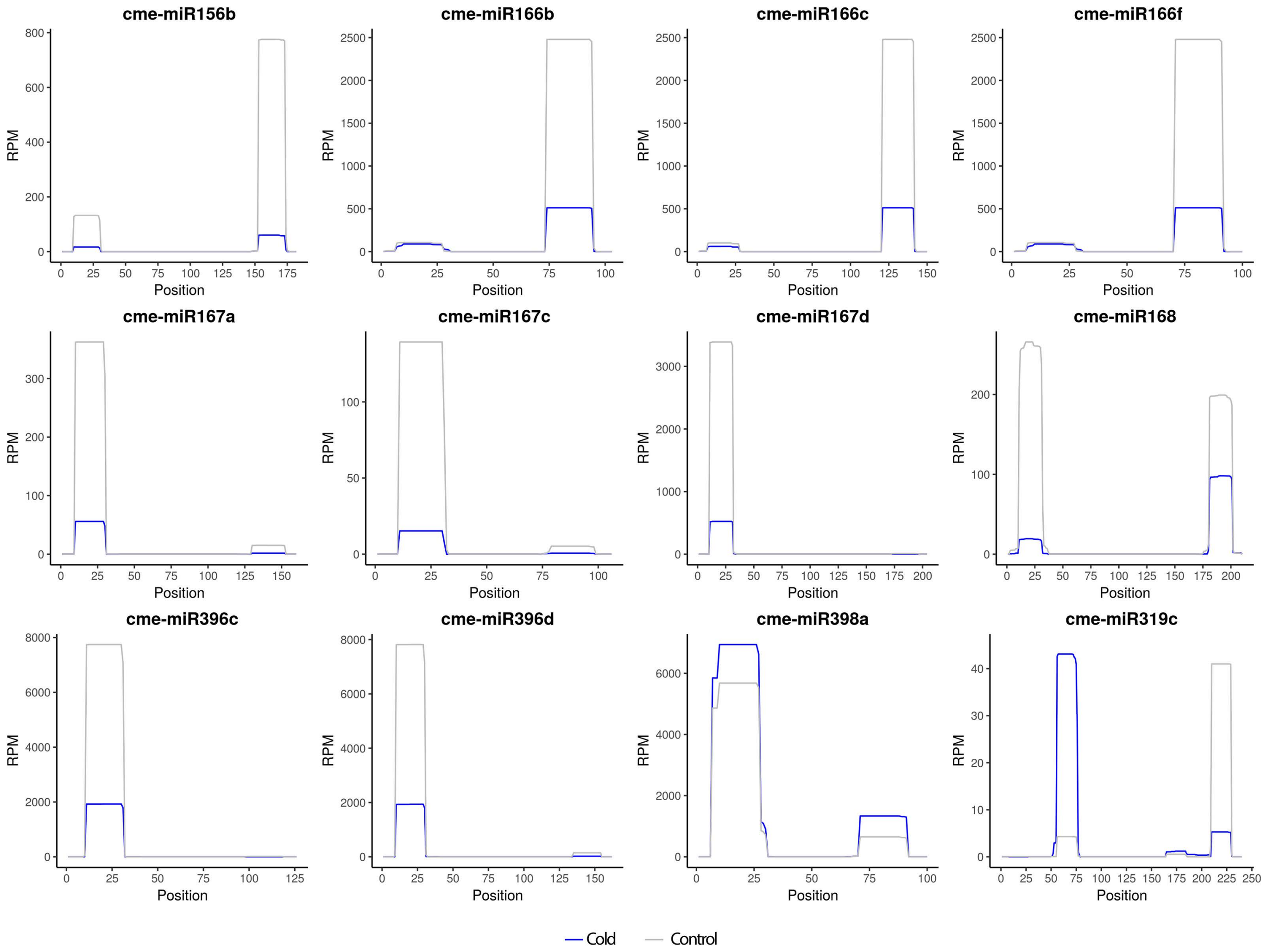

Figure S6: Alternative precursors processing induced by cold is a pri-miR319c specific phenomenon. The sRNAs (ranging 20 nt to 25 nt) recovered from cold-exposed (blue line) and control (gray line) melon plants were plotted (allowing only exact matching) onto the precursors sequences of cold-responsive miRNAs detected in melon. The values on the Y-axis represent the number of total reads in each library (normalized in reads per million). The nucleotide positions of the diverse pri-miRNA sequences analyzed are represented on the X-axis. In the figure could be observed that only pri-miR319c exhibits antagonist plot accumulation (a plot higher in cold and other higher in control) that evidence alternative processing. The rest of analyzed pri-miRNAs shown a comparable plots relation in both cold-exposed and control samples. Only peaks containing more that 5 RPM are showed.

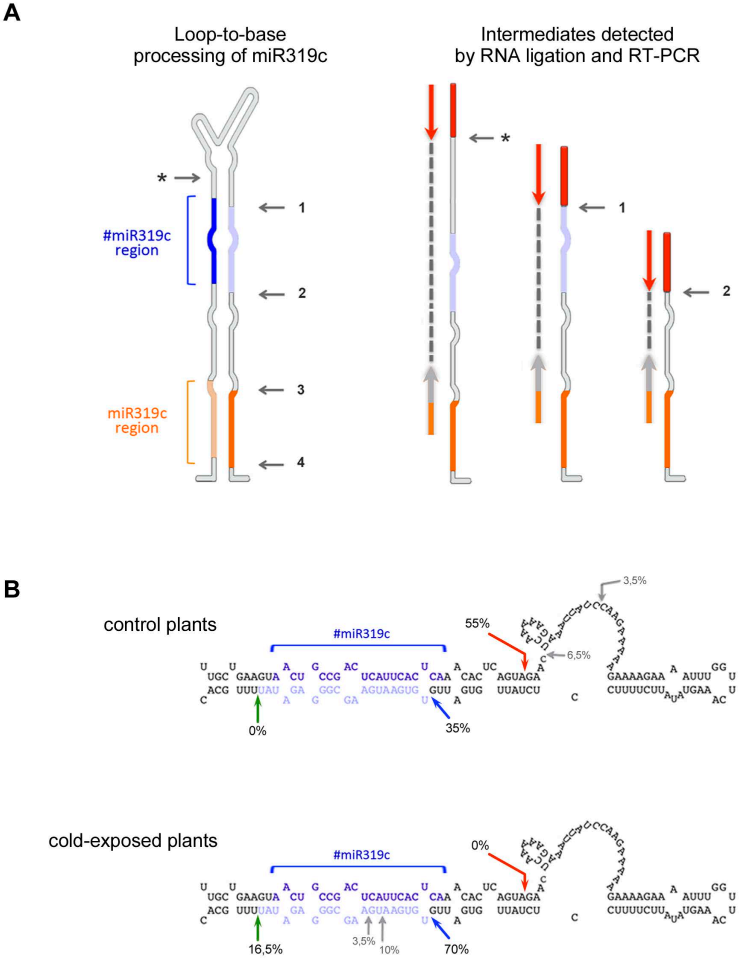

Figure S7: Specific processing of upper-stem of miR319c precursor estimated by 5´RACE. A, Scheme illustrating the method used to identify processed precursor intermediates and the expected fragments. (*) Indicates the partial cut in the 5´arm. (1 to 4) Represents the four-cleavage reaction expected for canonical loop-to-base-processing of miR319c precursor. Arrows indicate the position of the oligos used in the last PCR amplification. **B**, Schemes showing the predicted secondary structure (estimated from sequence >METC022194) and the abundance (in percentage of 30 analyzed sequences) of the cleavage sites detected in processed miR319c precursor recovered from control (upper panel) and cold exposed (lower panel) plants. Red arrow shows the partial processing point at 5´arm. Blue and green arrows indicate processing points 1 and 2, respectively. Gray arrows show the less abundant and unspecific cleavage site.

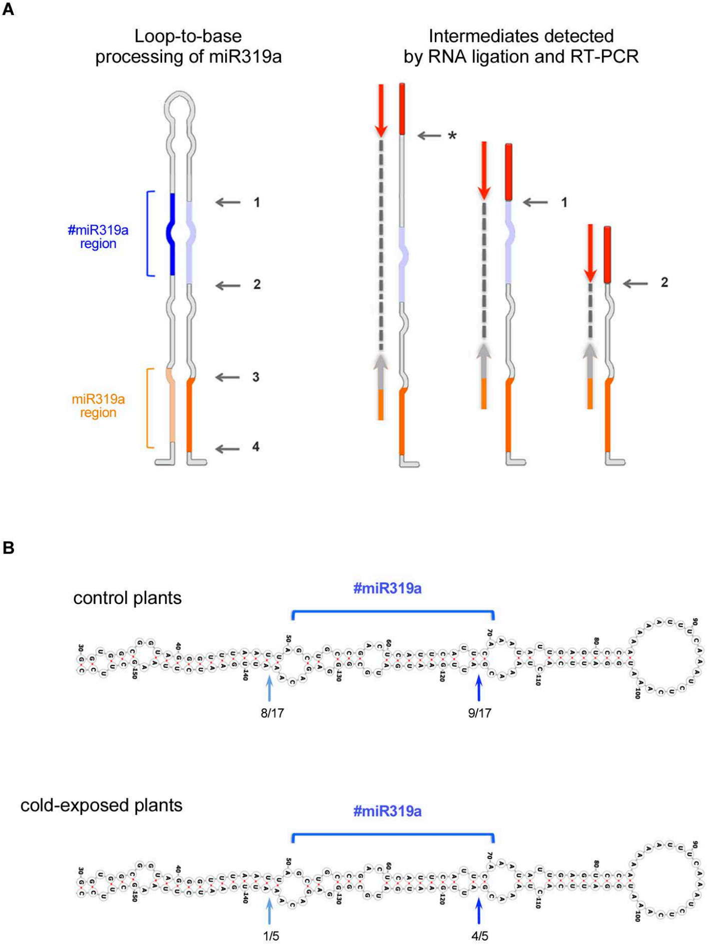

Figure S8: Processing of miR319a precursor in melon, estimated by 5´RACE. A, Scheme illustrating the method used to identify processed precursor intermediates and the expected fragments. Represents the four-cleavage reaction expected for canonical loop-to-base-processing of miR319A precursor. Arrows indicate the position of the oligos used in the last PCR amplification. **B**, Schemes showing the predicted secondary structure and the abundance (in numbers) of the cleavage sites detected in processed miR319a precursor recovered from control (upper panel) and cold exposed (lower panel) plants. Blue and magenta arrows indicate the processing point 1 and 2. 15 and 5 sequences were respectively analyzed for precursors recovered from control and cold-exposed plants.

	miRNA sequence	Target	Expect.	UPE	miRNA start	miRNA end	Target start	Target end	Target Description	Inhibition	miRNA/target aligned fragment
Schema V1 (2011)											
	CTTGGACTGAAGGGAGCTCCT	MU58965	3.5	17.908	1	21	257	277	Cucumis melo transcription factor TCP2-like	Cleavage	miRNA 21 UCCUCGAGGGAAGUCAGGUUC 1<br>:::.: :::::::::::::<br>Target 257 AGGGGGACCCUUCAGUCCAAU 277
	CTTGGACTGAAGGGAGCTCCT	MU58222	4.0	20.26	1	21	157	177	Cucumis melo transcription factor TCP4	Cleavage	miRNA 21 UCCUCGAGGGAAGUCAGGUUC 1<br>:::.: ::::::::::::.<br>Target 157 AGGGGACCCCUUCAGUCCAGU 177
Schema V2 (2017)											
	CTTGGACTGAAGGGAGCTCCT	MU58965	4.0	-1.0	1	21	257	277	Cucumis melo transcription factor TCP2-like	Cleavage	miRNA 21 UCCUCGAGGGAAGUCAGGUUC 1<br>:::.: :::::::::::::<br>Target 257 AGGGGGACCCUUCAGUCCAAU 277

Figure S9: TCP2 is the primary target for miR319c activity. Details of the target prediction for miR319c performed with psRNA Target versions V1 and v2. Limit for higher allowed expectative was established in 5.

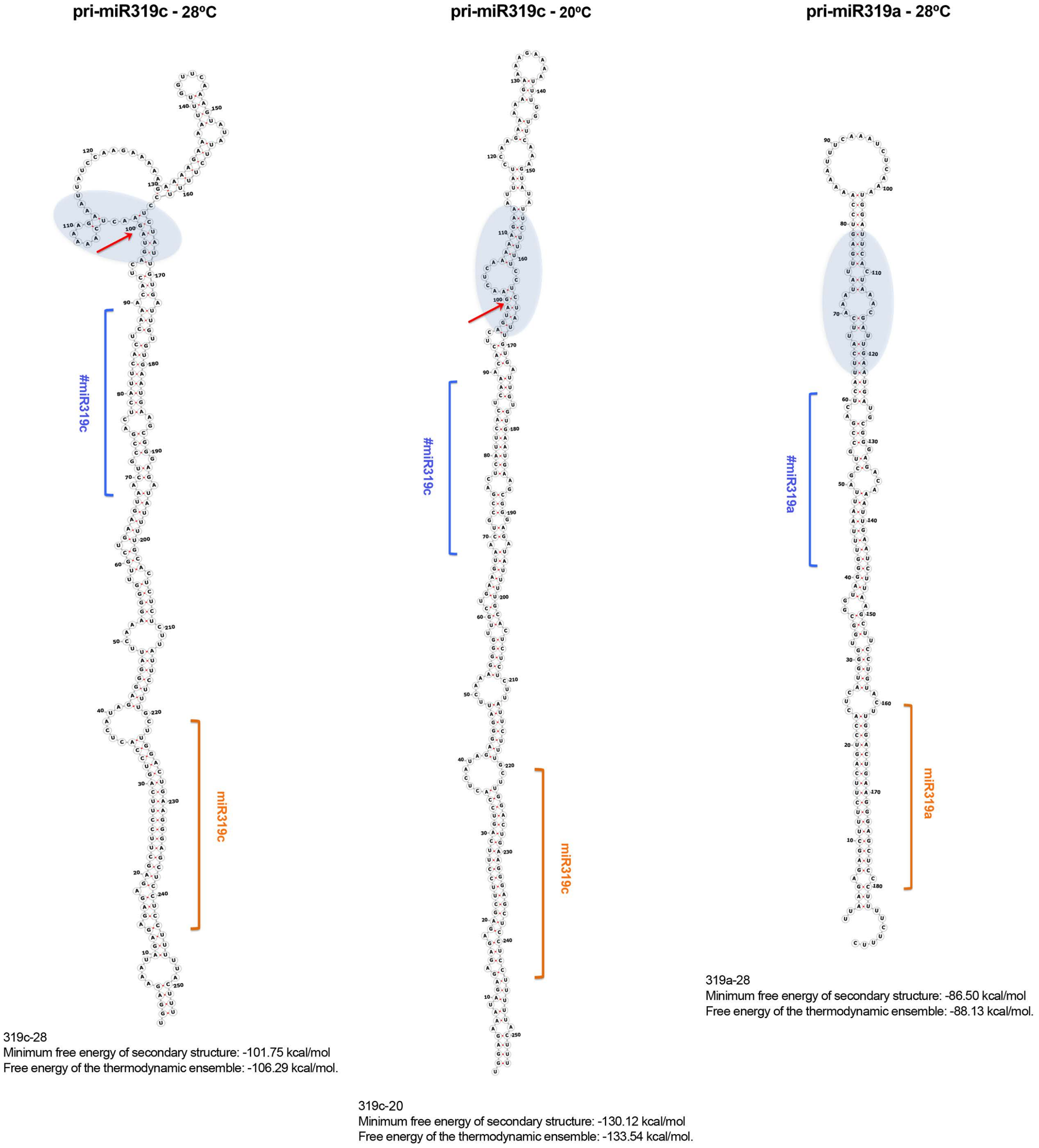

Figure S10: The secondary structure of miR319c precursors is temperature-dependent. Schemes showing the secondary structure predicted for miR319c precursor at 28ºC (left panel) and 20 ºC (central panel). As is shown in the figure, the secondary structure is strongly modified when is predicted establishing a lower folding temperature. The hairpin predicted for miR319c in cold condition mimic the secondary structure of miR319a precursor (right panel). Gray shadow indicate the unstructured domain near to partial cleavage position in the 5´arm of miR319c hairpin at 28ºC. Homologous regions are also detailed for precursors of miR319c at 20ºC and miR319a. Red arrow shows the partial processing point at 5´arm of pri-miR319c. Values of RNAfold structure prediction are also included below each secondary structure.

Table S1: Description of sRNA sequences fully homologous to cme-miR319 family members.

sRNA sequence	lenght	pri-miR319	arm	log2FC	logCPM	FDR
TGGACTGAAGGGAGCTCCCTT	21	***a***	3'	-4,093	2,336	< 1x10-5
TTGGACTGAAGGGAGCTCCCTT	22	***a***	3'	-5,585	5,201	< 1x10-5
TTGGACTGAAGGGAGCTCCC	20	***a/b***	3'	-3,88	6,882	< 1x10-5
CTTGGACTGAAGGGAGCTCCCT	22	***a/b***	3'	-3,46	5,043	< 1x10-5
TTGGACTGAAGGGAGCTCCCT	21	***a/b***	3'	-4,305	8,185	< 1x10-5
CTTGGACTGAAGGGAGCTCCC	21	***a/b***	3'	-5,12	3,209	< 1x10-5
TGGACTGAAGGGAGCTCCCT	20	***a/b***	3'	-3,673	2,775	< 1x10-5
TTGGACTGAAGGGAGCTCCCTC	22	***b***	3'	-3,74	3,916	< 1x10-5
CTTGGACTGAAGGGAGCTCCT	21	***c***	3'	-6,702	2,156	< 1x10-5
TTGGACTGAAGGGAGCTCCT	20	***c***	3'	-3,482	5,703	< 1x10-5
ATTGGACTGAAGGGAGCTCCT	21	***d***	3'	-4,592	3,339	< 1x10-5
TTGGACTGAAGGGAGCTCCTTC	22	***d***	3'	-1,643	2,399	0,002
ATTGGACTGAAGGGAGCTCC	20	***d***	3'	-2,933	2,701	< 1x10-5

Table S2: sRNA sequences associated to cme-#miR319c.

sRNA sequence	lenght	pri-miR319		log2FC	logCPM	FDR
AACTGCCGACTCATTCACTCA	21	***c***	5'	2,807	4,492	< 1x10-5
AACTGCCGACTCATTCACTC	20	***c***	5'	2,49	3,732	< 1x10-5

Table S3: Detail of the primers used in this work

OLIGOS FOR 5'-RACE TARGETS	
5′ adapter	5′-CGACUGGAGCACAGGACACUGACAUGGACUGAAGGAGUAGAAA-3′
Oligo(dT)30 primer	5′-ATTCTAGAGGCCGAGGCGGCCGACATG-d(T)30-3′
5' GeneRacer primer	5′-AGGACACTGACATGGACTGAAGGAGTAG-3′
3' GeneRacer primer	5′-ATTCTAGAGGCCGAGGCGGCCGACATG-3′
TCP2-R2	5'- TGCTGGTGTCAACTGATGGTG -3'
TCP2-R3	5'- CCCTCTAAGTGAGGAAACCTCTGAAGG -3'

OLIGOS FOR qRT-PCR OF miR319 PRECURSORS	
miR319a-F	5'-GGGTTTAATTAGCTGCCGAC-3'
miR319b-F	5'-TCGGTTGTTACTTAGCTGCTG-3'
miR319c-F	5'-CTGAAGTAACTGCCGACTC-3'
miR319d-F	5'-CGTTGCTGCTCATTCGTTAG -3'
miR319-R	5'-GGAGCTCCCTTCAGTCCAA-3'

OLIGOS FOR qRT-PCR OF miR319 TARGETS	
TCP2-F	5'-TCAACATTTCTCGTTCGTCCC-3'
TCP2-R	5'-ATGCAGCTCCGATGAAAAGG-3'

OLIGOS FOR 5´- RACE PROCESSED PRECURSORS		
5´Gene Racer	5'-AGGACACTGACATGGACTGAAGGAGTAG-3'	
miR319c short-R1	5'- gcAAA GGA GGA GCT CCC TTC AG -3'	to detect cuts *, 1 and 2
miR319c short-R2	5'- cgg GCA AAG AAT AAG AGA GAG TGC -3'	
miR319c lenght-R1	5’-gggcggATAAATTTGGGGTTTGATAG-3’	to detect cuts *, and 1 to 4.
miR319c lenght-R2	5’-ggccgGATAGTTTTATGGGGAAAG-3’	
mir319a-R2	5’-gcg ccT ACA GGA AGC TTA AGA TTC-3’	to detect cuts *, 1 and 2

OLIGOS FOR qRT-PCR OF COLD RELATED TRANSCRIPTS	
HY5-F	5'- TCG TAT GCG CTC GAG TAG TG -3'
HY5-R	5'- TTT ACC TGG CGC TGA AAT GC -3'
CHS-F	5'- GCG CCG ATT ACC AAC TCC TC -3'
CHS-R	5'- GAC CAA CAC TCT GGC TCC TC -3'
CHI-F	5'- AGT TAG GGC GTT GGA GAT CG -3'
CHI-R	5'- TAA CTC CGC CGC ACT CTT TC -3'
